# Genotype–phenotype correlation of X-linked Alport syndrome observed in both genders: a multicenter study in South Korea

**DOI:** 10.1038/s41598-023-34053-7

**Published:** 2023-04-26

**Authors:** Ji Hyun Kim, Seon Hee Lim, Ji Yeon Song, Myung Hyun Cho, HyeSun Hyun, Eun Mi Yang, Jung Won Lee, Min Hyun Cho, Min Ji Park, Joo Hoon Lee, Jiwon Jung, Kee Hwan Yoo, Kyung Mi Jang, Ki Soo Pai, Jin-Soon Suh, Mee Kyung Namgoong, Woo Yeong Chung, Su Jin Kim, Eun Young Cho, Kyung Min Kim, Nam Hee Kim, Minsun Kim, Jin Ho Paik, Hee Gyung Kang, Yo Han Ahn, Hae Il Cheong

**Affiliations:** 1 Department of Pediatrics, Seoul National University Bundang Hospital, Seongnam, South Korea; 2 Department of Pediatrics, Seoul National University College of Medicine, Seoul, South Korea; 3 Department of Pediatrics, Pusan National University Yangsan Children’s Hospital and School of Medicine, Yangsan, South Korea; 4 Department of Pediatrics, Hallym University Sacred Heart Hospital, Anyang, South Korea; 5 Department of Pediatrics, College of Medicine, St. Vincent’s Hospital, The Catholic University of Korea, Seoul, South Korea; 6 Department of Pediatrics, Chonnam National University and School of Medicine, Gwangju, South Korea; 7 Department of Pediatrics, Ewha Womans University School of Medicine, Seoul, South Korea; 8 Department of Pediatrics, Kyungpook National University, School of Medicine, Daegu, South Korea; 9 Department of Pediatrics, Asan Medical Center Children’s Hospital, University of Ulsan College of Medicine, Seoul, South Korea; 10 Department of Nephrology, Woori Children’s Hospital, Seoul, South Korea; 11 Department of Pediatrics, Yeungnam University College of Medicine, Gyeongsan, South Korea; 12 Department of Pediatrics, Ajou University School of Medicine, Suwon, South Korea; 13 Department of Pediatrics, Bucheon St. Mary’s Hospital, College of Medicine, The Catholic University of Korea, Seoul, South Korea; 14 Department of Pediatrics, Wonju Severance Christian Hospital, Yonsei University Wonju College of Medicine, Wonju, South Korea; 15 WooYeong Chung Pediatrics Clinic, Busan, South Korea; 16 Department of Pediatrics, Inha University College of Medicine, Inha University Hospital, Incheon, South Korea; 17 Department of Pediatrics, Chungnam National University Hospital, Daejeon, South Korea; 18 Department of Pediatrics, Inje University Ilsan Paik Hospital, Goyang, South Korea; 19 Department of Pediatrics, Jeonbuk National University Medical School, Jeonju, South Korea; 20 Department of Pathology, Seoul National University Bundang Hospital and Seoul National University College of Medicine, Seongnam, South Korea; 21 Department of Pediatrics, Seoul National University Children’s Hospital, Seoul, South Korea; 22 Kidney Research Institute, Medical Research Center, Seoul National University, Seoul, South Korea; 23 Department of Pediatrics, Seoul Red Cross Hospital, Seoul, South Korea

**Keywords:** Medical research, Nephrology

## Abstract

The genotype–phenotype correlation of the X-linked Alport syndrome (XLAS) has been well elucidated in males, whereas it remains unclear in females. In this multicenter retrospective study, we analyzed the genotype–phenotype correlation in 216 Korean patients (male:female = 130:86) with XLAS between 2000 and 2021. The patients were divided into three groups according to their genotypes: the non-truncating group, the abnormal splicing group, and the truncating group. In male patients, approximately 60% developed kidney failure at the median age of 25.0 years, and kidney survival showed significant differences between the non-truncating and truncating groups (*P* < 0.001, hazard ratio (HR) 2.8) and splicing and truncating groups (*P* = 0.002, HR 3.1). Sensorineural hearing loss was detected in 65.1% of male patients, while hearing survival periods showed a highly significant difference between the non-truncating and truncating groups (*P* < 0.001, HR 5.1). In female patients, approximately 20% developed kidney failure at the median age of 50.2 years. The kidney survival was significantly different between the non-truncating and truncating groups (*P* = 0.006, HR 5.7). Our findings support the presence of genotype–phenotype correlation not only in male patients but also in female patients with XLAS.

## Introduction

Alport syndrome (AS) is a progressive inherited nephropathy that leads to kidney failure and is accompanied by sensorineural hearing loss (SNHL) and ocular abnormalities, such as lenticonus and fleck retinopathy^1,2^. Although previous studies suggest a prevalence of approximately 1/50,000 for AS, a recent publication reported significantly higher estimated frequencies of predicted pathogenic *COL4A4*-*COL4A5* variants^2^. While the most common form of AS inheritance is the autosomal dominant form, which affects about 1 in 106 individuals, the X-linked AS (XLAS) form, which is a more severe phenotype, affects approximately 1/2320 of the population^2^.

In male patients, 90% develop chronic kidney disease (CKD) with a glomerular filtration rate (GFR) category 5 (GFR < 15 ml/min per 1.73 m^2^ or treatment by kidney replacement) (CKD G5)^3^ by the age of 40 years, with a median age of 25 years^4^, and exhibit a very strong genotype–phenotype correlation. For example, truncating variants (rearrangement, nonsense, and frameshift) show a severe phenotype that leads to the earlier development of kidney failure and hearing loss, whereas non-truncating variants (missense and in-frame variants) present a milder phenotype. In the case of splice variants, the overall phenotype was intermediate^4–6^. The development of hearing loss and ocular changes showed a trend similar to that of kidney prognosis^4,5,7^. In female patients with XLAS, no genotype–phenotype correlations have been observed as they generally present favorable outcomes, with approximately 25% of cases progressing to CKD G5 throughout their lifetime, with a median age of 65 years^8,9^. However, a recent systematic review article^10^ reported a correlation between severe genotypes and proteinuria. Missense variants are the most common (60%) variants in male patients, and the overall phenotype is favorable, but the prognosis is variable, ranging from mild to severe^11^. The majority (about 85%) of missense variants are found in the intermediate collagenous domain, and glycine (Gly) substitutions constitute 95% of these variants (in ARUP, arup.utah.edu/database/)^11^. Glycine substitution adjacent to a noncollagenous (NC) interruption site is known to often be hypomorphic with a milder phenotype^11,12^, and other mitigating factors associated with variant location and affected residue have been suggested^2^. Histologically, the positive expression of the type IV collagen α5 chain in immunofluorescence staining tends to show a milder phenotype^12^. Here, for the first time, we analyzed the genotype–phenotype correlation in Korean patients of both sexes with XLAS.

## Patients and methods

### Study participants and definitions

This retrospective study was conducted at 12 medical centers in Korea. A total of 124 probands (85 males and 39 females) diagnosed with XLAS between September 2000 and June 2021 were enrolled. The diagnosis of XLAS was primarily based on the detection of pathogenic *COL4A5* variants. Patients with variants classified as having uncertain significance were enrolled if the kidney biopsy findings were consistent with AS. Clinical and laboratory data were obtained through a review of electronic medical records. In addition, some data analyses included the available clinical data of 92 (45 males and 47 females) affected family members with the same *COL4A5* variants as the proband. Therefore, the number of analyzed subjects differed for each parameter because of the missing variable data of the affected family members. All patients and their affected family members analyzed in this study were Korean.

The estimated GFR (eGFR) was calculated using the creatinine-cystatin C-based Schwartz 2012 equation^13^ for patients under 18 years of age and the CKD Epidemiology Collaboration 2021 formula^14^ for patients aged 18 years and older. CKD stage was determined using the Kidney Disease: Improving Global Outcomes (KDIGO) CKD classification^3^. Asymptomatic urinary abnormalities (AUA) were defined as the presence of microscopic hematuria and/or proteinuria, detected incidentally during school screening or routine laboratory findings during hospital visits, without any accompanying subjective symptoms. Nephrotic syndrome (NS) was defined as nephrotic-range proteinuria (first morning urine protein to creatinine ratio (UPCR) ≥ 2.0 mg/mg or ≥ 3 + dipstick) and serum albumin < 3.0 g/dL according to the KDIGO 2021 guideline^15^. The occurrence of hearing impairment was defined as subjective symptoms or according to pure tone audiometry results based on the World Health Organization classification^16^. Patients with other potential causes of hearing impairment, such as ear infections or medications, were excluded from the analysis. Ocular lesions included maculopathy, lens abnormalities, corneal opacities, cataracts, myopia, keratoconus, and astigmatism^7,17,18^.

### Ethical approval

This study was approved by the institutional ethics committee of each hospital, including the Seoul National University Bundang Hospital, Seoul National University Children’s Hospital, Asan Medical Center, Chonnam National University Hospital, Chungnam National University Hospital, Ewha Womans University Hospital, Inha University Hospital, Korea University Hospital, Kyungpook National University Hospital, Pusan National University Yangsan Hospital, the Catholic University of Korea, Bucheon St. Mary’s Hospital, and Wonju Severance Christian Hospital, which waived the need for informed consent because only retrospective study data was collected. All procedures were performed in accordance with the Declaration of Helsinki.

### Mutational analysis

Genetic tests were performed using Sanger sequencing of *COL4A5* in most probands, except for four female probands in which targeted exome sequencing (TES) (three patients) and whole-exome sequencing (WES) (one patient) were used. Family members were screened using Sanger sequencing if they presented with clinical symptoms related to AS, such as hematuria, proteinuria, CKD, or hearing loss. The detailed procedures of Sanger sequencing^19^, TES^20^, and WES^21^ have been described in previous literature. Sequences were compared with the reference mRNA sequences (GenBank Accession: NM_000495.4). The interpretation of variants followed the American College of Medical Genetics and Genomics and the Association for Molecular Pathology guidelines, and variants classified as pathogenic or likely pathogenic were considered disease-causing variants (Supplementary Table S1)^22^. In addition, we included some variants classified as having uncertain significance if the kidney biopsy findings were consistent with AS and a definite family history of kidney disease or hearing loss was present (Supplementary Table S2). The novelty of the variants was determined by referring to the ClinVar, Varsome, LOVD, and HGMD databases.

According to the genotypes, the patients were divided into three groups: Group 1, missense variants or short in-frame deletions, insertions, or duplications; Group 2, abnormal splicing variants; and Group 3, nonsense variants, frame-shifting variants, or large deletions encompassing one or more exons.

### Statistical analyses

Statistical analyses were performed using SPSS software, version 20.0 (IBM Corp., Armonk, NY, USA). Descriptive statistics, including the median (interquartile range [IQR]) or frequency counts (percentage), were used to describe the sample population. Categorical variables were analyzed using the Chi-square test or Fisher’s exact test. Continuous variables were compared using the Kruskal–Wallis test or the Mann–Whitney U test. The Kaplan–Meier method and the log-rank statistic were used to present the occurrence of events (age at CKD G5 and age at detection of SNHL). The hazard ratio (HR) was analyzed using the Cox proportional hazards model. Robust statistical tests were used for the comparison between groups and intrafamilial correlations^5^. A *P*-value of < 0.05 was considered significant.

## Results

### Overall manifestations of the patients

#### Kidney phenotypes

The detailed clinical kidney presentations and outcomes are summarized in Table 1, and the phenotypes and genotypes of the individual patients are listed in Supplemental Table S1. The median age of onset and age at the last visit were 4.4 and 20.8 years, respectively. The most common initial presentation was AUA, followed by macroscopic hematuria, in both males and females. While no female patients presented with NS, approximately 20% of male patients presented with the condition, along with hematuria, at the median onset age of 7.0 (IQR 3.5–10.3) years.

**Table 1:** Overall kidney phenotypes of the X-linked Alport syndrome patients.

Kidney and extrarenal phenotypes	Male patients, analyzed	Female patients, analyzed	Total
Onset age, years	4.4 (2.7–9.7), n = 98	4.3 (2.3–10.0), n = 60	4.4 (2.5–10.0), n = 158
Age at last visit, years	22.2 (15.8–28.8), n = 90	19.1 (10.0–31.3), n = 60	20.8 (13.6–29.2), n = 150
Onset presentation	n = 94	n = 64	n = 158
AUA	38 (40.4)	41 (64.1)	79 (50.0)
Macroscopic hematuria	31 (33.0)	21 (32.8)	52 (32.9)
Nephrotic syndrome	18^a^ (19.2)	0	18 (11.4)
Chronic kidney disease	7 (7.5)	2 (3.1)	9 (5.7)
Progression of kidney phenotypes			
Hematuria	123/123 (100)	63/63 (100)	186/186 (100)
Overt proteinuria^b^	122/123 (99.2)	58/63 (92.1)	180/186 (96.8)
Macroscopic hematuria	49/85 (57.7)	30/46 (65.2)	79/131 (60.3)
Chronic kidney disease G5	70/121 (57.9)	14/68 (20.6)	84/189 (44.4)
Median kidney survival age, years (95% CI)^c^	25.0 (23.5–26.5), n = 120	50.2 (39.0–61.5), n = 68	29.5 (25.0–34.0), n = 188
Use of RASi	85/87 (97.7)	52/59 (88.1)	137/146 (93.8)
Age starting RASi, years	9.6 (5.9–14.5), n = 82	9.5 (3.1–19.2), n = 49	9.6 (5.2–15.7), n = 131
Extrarenal phenotypes			
Sensorineural hearing loss	71/109 (65.1)	10/71 (14.1)	81/180 (45.0)
Median hearing survival age, years (95% CI)^c^	19.9 (13.8–26.0), n = 89	53.4 (47.5–59.2), n = 56	32.0 (5.9–43.5), n = 145
Ocular abnormalities	20/63 (31.7)	2/21 (9.5)	22/84 (26.2%)

Values are presented as median (interquartile range) or numbers (%).

The number of analyzed subjects differed for each parameter because of the missing variable data of the affected family members.

AUA, asymptomatic urinary abnormality; RASi, renin-angiotensin system inhibitor; CI, confidence interval.

^a^Three patients presented with nephrotic syndrome and macroscopic hematuria.

^b^Urine protein to creatinine ratio > 0.2 mg/mg.

^c^It is estimated by Kaplan–Meier analysis.

In male patients, UPCR at the initial hospital visit at a median age of 8.9 (IQR 4.1–14.3) years was 1.6 mg/mg (IQR 0.5–4.0 mg/mg), and during follow-up, almost all patients except for one developed overt proteinuria (UPCR > 0.2 mg/mg). More than 90% of female patients developed overt proteinuria during follow-up. All patients exhibited hematuria. Treatment with renin-angiotensin system inhibitors (RASi) was initiated in all male patients at a median age of 9.5 years, except for two patients already in CKD G5 at the initial presentation. Nearly 90% of the female patients started taking RASi at the same median age as the male patients.

**Figure 1:**
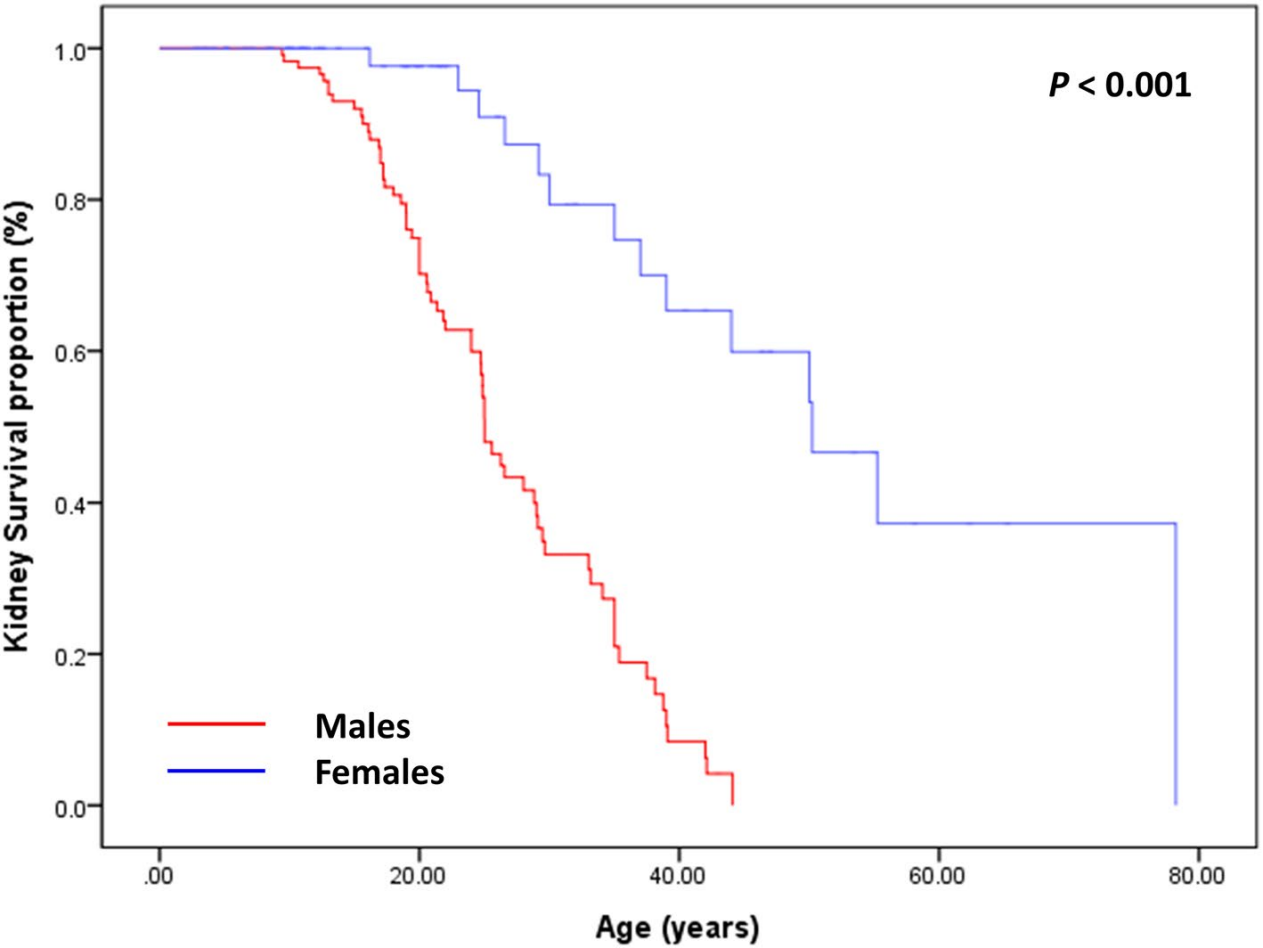
Kidney survival in 188 patients (120 males, 68 females) with X-linked Alport syndrome according to sex.

Approximately 60% of male patients had reached CKD G5 at the median age of 25.0 ([95% confidence interval (CI)] 23.5–26.5) years, which was estimated by the Kaplan–Meier method (Fig. 1), and nearly 90% had reached CKD G5 by 39 years. In female patients, approximately 20% progressed to kidney failure at the median age of 50.2 (95% CI 39.0–61.5) years, as estimated by Kaplan–Meier analysis, and approximately 30% and 60% reached CKD G5 by 37 and 55 years, respectively (Fig. 1).

Kidney biopsy was performed in 52.3% (68 of 130 patients) of male patients at a median age of 10.4 (IQR 7.1–14.3) and 41.9% (36 of 86 patients) of female patients at a median age of 10.6 (IQR 7.0–22.9) years. The pathological diagnosis was AS in 59 (86.8%) male and 20 (55.6%) female patients, while the remaining patients were diagnosed with immunoglobulin A nephropathy, mesangial proliferative glomerulonephritis, or thin basement membrane disease. On the second biopsy, AS was diagnosed in four male and three female patients.

The expression of the type IV collagen α5 chain was examined by immunofluorescence microscopy in 23 male and 17 female patients. In male patients, the expression was completely absent in 17 (73.9%), weak in 3 (13.0%), and normal in 3 (13.0%) patients. In female patients, 8 (47.1%) showed a mosaic pattern, 7 (41.2%) showed a normal pattern, and 2 (11.8%) showed a total loss.

#### Extrarenal phenotypes

In male patients, 65.1% developed SNHL at the median age (hearing survival age) of 19.9 (95% CI 13.8–26.0) years, while 14.1% of female patients developed SNHL at the median age of 53.4 (95% CI 47.5–59.2) years, which was estimated by Kaplan–Meier analysis (Table 1). A total of 47.9% (34/71; no data available for 25 patients) of male patients and 40% (4/10; no data available for five patients) of female patients with SNHL were recommended for or wore hearing aids at the median ages of 11.3 (IQR 9.9–16.7) and 45.9 (IQR 33.8–50.8) years, respectively. Ocular examination records were available for 63 males and 21 females. Twenty (31.7%) male patients and two (9.5%) female patients showed ocular abnormalities such as anterior lenticonus or fleck retinopathy.

#### Genotypes

Among the 124 probands, 56 (45.2%) had missense variants, 23 (18.5%) had abnormal splicing, 19 (15.3%) had nonsense variants, 21 (16.9%) had short frame-shifting insertion or deletion variants, and 5 (4.0%) had other types of variants (Table 2 and Supplemental Table S1). Among the 121 different variants, 93 (76.9%) were novel. Two variants (p.Gly400Val and p.Arg1677Ter) were common in three families, and two other variants (c.466-2A>G and c.991-7T>A) were detected in both families. One male proband (P49 in Supplemental Table S1) had two *COL4A5* variants, p.Gly1057Glu and p.Gly1442Cys.

**Table 2:** Genotype groups of the X-linked Alport syndrome patients.

Genotypes	Number of the male patients (probands)	Number of the female patients (probands)	Number of the total patients (probands)
Group 1	61 (39)	40 (19)	101 (58)
Missense variants	60 (38)	38 (18)	98 (56)
In-frame short deletion	1 (1)	2 (1)	3 (2)
Group 2			
Abnormal splicing variants	30 (16)	19 (7)	49 (23)
Group 3	39 (30)	27 (13)	66 (43)
Nonsense variants	12 (9)	15 (10)	27 (19)
Frame-shifting short insertion or deletions	23 (18)	11 (3)	34 (21)
Large deletions	4 (3)	1 (0)	5 (3)
Total	130 (85)	86 (39)	216 (124)

### Genotype–phenotype correlations

#### Male patients

##### Kidney phenotypes

At the time of onset, NS was significantly more prevalent (*P* = 0.023, odds ratio [OR] 3.3), and AUA was significantly less prevalent (*P* = 0.043, OR 0.4) in Group 3 patients when compared to Group 1 or 2 patients (Table 3).

**Table 3:** Genotype–phenotype correlations of male patients with X-linked Alport syndrome.

Kidney and extrarenal phenotypes	Group 1	Group 2	Group 3	*P* value
Onset age, years	4.1 (2.7–9.6)	5.1 (1.6–22.9)	4.8 (2.8–8.7)	0.987
Age at last visit, years	22.3 (15.6–28.5)	19.4 (14.8–31.9)	22.8 (17.7–26.9)	0.998
Onset presentation				
AUA	20 (48.8)	10 (45.5)	8 (25.8)^b,c^	0.121
Macroscopic hematuria	14 (34.2)	6 (27.3)	11 (35.5)	0.850
Nephrotic syndrome	4 (9.8)	4 (18.2)	10 (32.3)^b,c^	0.056
Chronic kidney disease	3 (7.3)	2 (9.1)	2 (6.5)	1.000
Total	41 (100)	22 (100)	31 (100)	
UPCR at initial visit (mg/mg)	1.5 (0.4–3.4)	0.9 (0.2–4.8)	2.0 (0.8–4.2)	0.413
Progression of kidney phenotypes				
Hematuria	56/56	29/29	38/38	1.000
Overt proteinuria*	56/56	28/29	38/38	1.000
Macroscopic hematuria	20/35 (57.4)	10/19 (52.6)	19/31 (61.3)	0.817
Chronic kidney disease G5	28/55 (50.9)	17/29 (58.6)	25/37 (67.6)	0.302
Median kidney survival age, years (95% CI)**	29.1 (23.5–34.7)	29.5 (21.4–37.6)	20.9 (15.4–26.3)^b,c^	0.001
Use of RASi (n/n)	37/38	19/19	29/30	1.000
Age starting RASi, years	10.5 (4.9–16.2)	9.6 (5.5–16.1)	8.0 (5.0–10.7)	0.174
Extrarenal phenotypes				
Sensorineural hearing loss	24/50 (48.0)^b^	19/27 (70.4)	28/32 (87.5)	< 0.001
Median hearing survival age, years (95% CI)**	31.8 (26.1–37.6)^a,b^	18.6 (9.6–27.6)	10.5 (9.1–12.0)	< 0.001
Ocular abnormalities	1/24 (4.2)^a,b^	8/22 (36.4)	11/19 (57.9)	< 0.001

Values are presented as median (interquartile range) or numbers (%).

The number of analyzed subjects differed for each parameter because of the missing variable data of the affected family members.

AUA, asymptomatic urinary abnormality; UPCR, urine protein-to-creatinine ratio (mg/mg); RASi, renin-angiotensin system inhibitors; CI, confidence interval.

*Urine protein-to-creatinine ratio > 0.2 mg/mg.

**It is estimated by Kaplan–Meier analysis.

^a^*P* value < 0.05 between Group 1 and Group 2.

^b^*P* value < 0.05 between Group 1 and Group 3.

^c^*P* value < 0.05 between Group 2 and Group 3.

**Figure 2:**
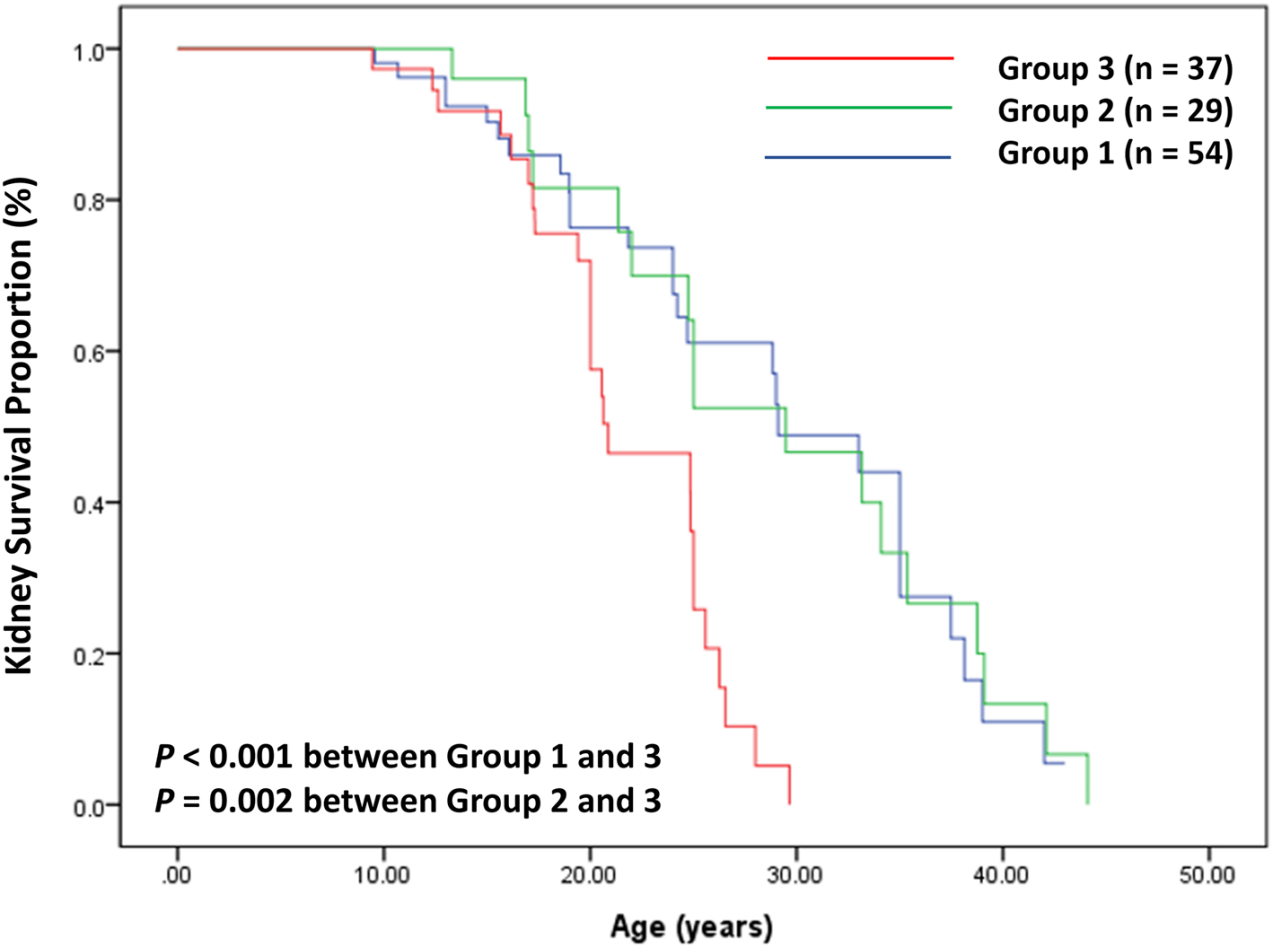
Kidney survival in 120 male patients with X-linked Alport syndrome according to the variant types of *COL4A5.*

When stratified according to variant types, approximately 50% of Group 1 patients and 70% of Group 3 patients developed CKD G5 at the median age (kidney survival periods) of 30 and 20 years, respectively. The kidney survival period showed a significant difference between Group 1 and Group 3 (*P* < 0.001, HR 2.8) and between Group 2 and Group 3 (*P* = 0.002, HR 3.1), which was estimated by Kaplan–Meier analysis. However, there was no difference between Groups 1 and 2. The kidney outcomes according to variant type are described in Table 3 and Fig. 2.

Of the 40 different Gly substitution variants, nine were located between exons 1–20, 29 between exons 21–47, and two in the NC domain. The location of Gly substitution variants (exons 1–20 versus 21–47) did not affect the severity of the kidney phenotypes. Furthermore, the difference in the amino acid substitution for Gly did not correlate with the kidney phenotypes (data not shown).

In 15 families, there were at least two (between two and four) male patients who progressed to CKD G5. Intrafamilial homogeneity in CKD progression (less than 15 [0–10.3] years of age at CKD G5) was observed in 13 families. Conversely, intrafamilial heterogeneity (15 or more [between 16 and 22.9] years of age at CKD G5) was observed in the remaining two families.

##### Extrarenal phenotypes

**Figure 3:**
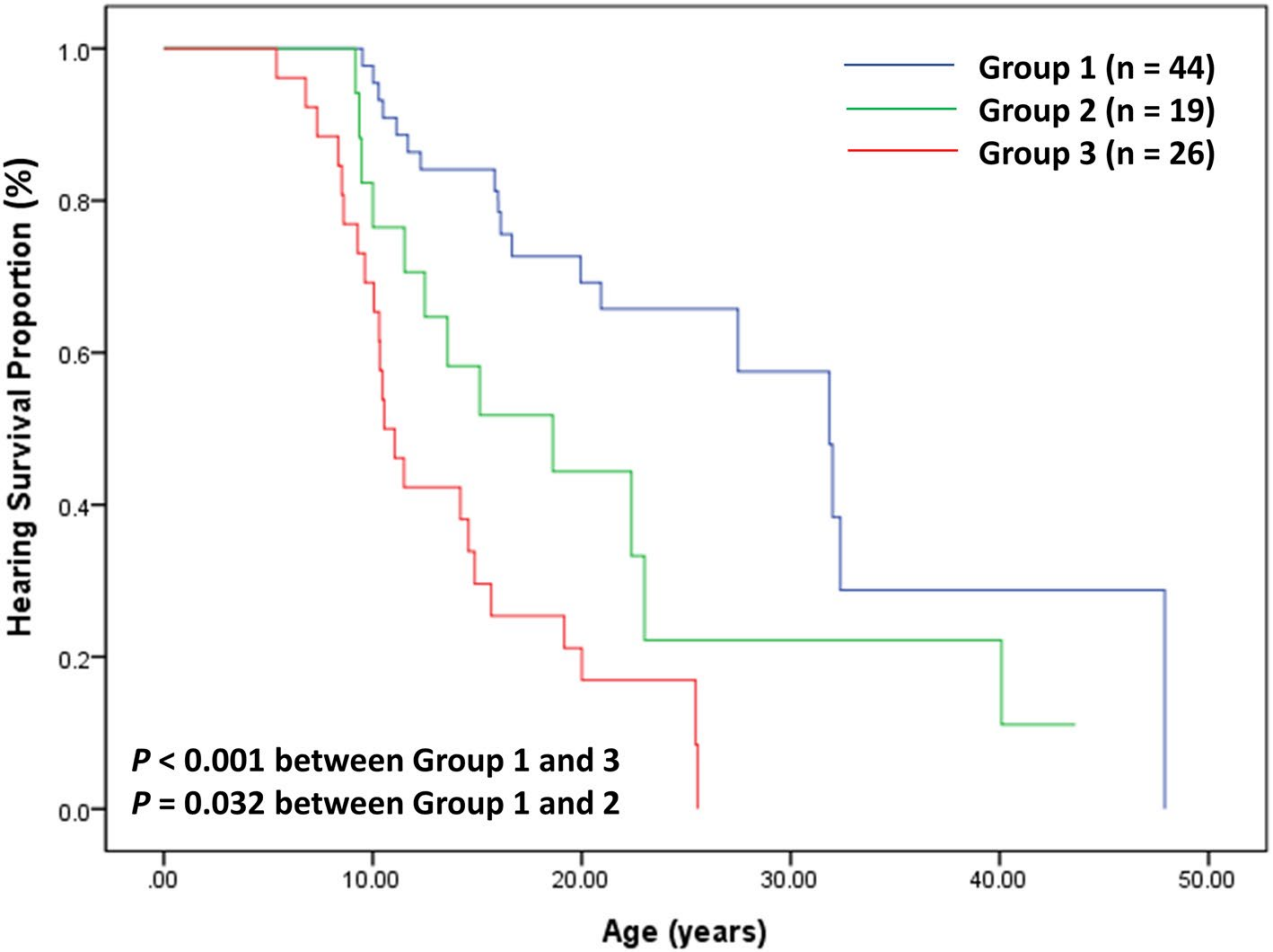
Hearing survival in 89 male patients with X-linked Alport syndrome according to the variant types of *COL4A5.*

SNHL was detected more commonly in Group 3 patients when compared to Group 1 patients (*P* < 0.001, OR 7.6 [95% CI 2.3–24.8]) (Table 3). Kaplan–Meier analyses estimated that the hearing survival period was significantly shorter in Group 3 than in Group 1 (*P* < 0.001, HR 5.1) and in Group 2 than in Group 1 (*P* = 0.032, HR 2.2). However, there was no difference between Groups 2 and 3. (Fig. 3) Ocular abnormalities were also more prevalent in Group 3 than in Group 1 (*P* < 0.001) and in Group 2 than in Group 1 (*P* < 0.001) (Table 3).

#### Female patients

##### Kidney phenotype

**Figure 4:**
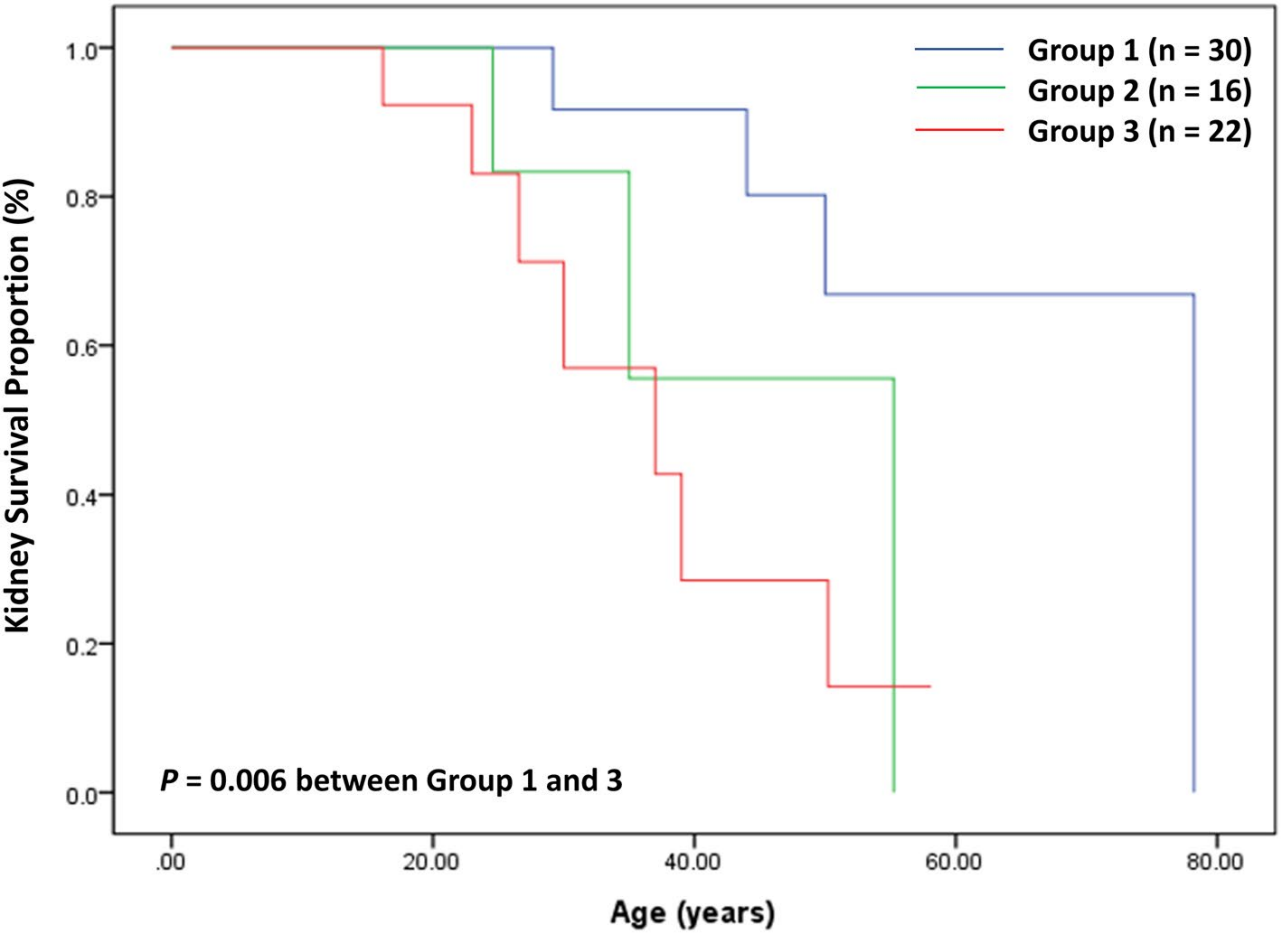
Kidney survival in 68 female patients with X-linked Alport syndrome according to the variant types of *COL4A5.*

The age of onset was lower in Group 3 than in Group 1 (*P* = 0.029) and Group 2 (*P* = 0.005), and Group 3 patients presented AUA less frequently than other groups (*P* = 0.037) (Table 4). Fourteen (20.6%) of the 68 female patients developed CKD G5. Group 3 patients developed CKD G5 significantly earlier than Group 1 (*P* = 0.006, HR 5.7), as well as Groups 1 and 2 (*P* = 0.013, HR 2.7) (Table 4 and Fig. 4).

**Table 4:** Genotype–phenotype correlations of female patients with X-linked Alport syndrome.

Kidney and extrarenal phenotypes	Group 1	Group 2	Group 3	*P* value
Onset age, years	5.4 (2.4–16.1)	7.6 (4.1–12.2)	2.4 (1.4–4.4)^a,b^	0.018
Age at last visit, years	20.1 (10.2–40.9)	20.3 (11.9–26.4)	14.2 (9.1–24.4)	0.217
Onset presentation				
AUA	21 (65.6)	13 (86.7)	7 (41.2)^b^	0.020
Macroscopic hematuria	10 (31.3)	2 (13.3)	9 (52.9)^b^	0.064
Chronic kidney disease	1 (3.1)	0	1 (5.9)	1.000
Total	32	15	17	
Progression of kidney phenotypes				
Hematuria	33/33 (100)	15/15 (100)	17/17 (100)	1.000
Overt proteinuria*	29/30 (96.7)	10/13 (76.9)	19/20 (95.0)	0.120
Macroscopic hematuria	14/21 (66.7)	4/9 (44.4)	12/16 (75.0)	0.367
Chronic kidney disease G5	4/30 (13.3)	3/16 (18.8)	7/22 (31.8)	0.318
Median kidney survival age, years (95% CI)**	78.2 (NE)	55.3 (NE)	37.0 (19.8–54.2)^a^	0.022
Use of RASi	26/29 (89.7)	10/13 (76.9)	16/17 (94.1)	0.436
Age starting RASi, years	10.2 (4.7–30.8)	10.0 (7.4–15.7)	5.0 (2.5–10.9)	0.133
Extrarenal phenotypes				
Sensorineural hearing loss	4/33 (12.1)	2/15 (13.3)	4/23 (17.4)	0.904
Median hearing survival age, years (95% CI)**	55.0 (51.5–58.5)	42.2 (NE)	NE	0.349
Ocular abnormalities	2/10 (20)	0/6 (0)	0/5 (0)	0.476

Values are presented as median (interquartile range) or numbers (%).

The number of analyzed subjects differed for each parameter because of the missing variable data of the affected family members.

AUA, asymptomatic urinary abnormality; RASi, renin-angiotensin system inhibitors; CI, confidence interval; NE, not estimated.

*Urine protein-to-creatinine ratio > 0.2 mg/mg.

**It is estimated by Kaplan–Meier analysis.

^a^*P* value < 0.05 between Group 1 and Group 3.

^b^*P* value < 0.05 between Group 2 and Group 3.

##### Extrarenal phenotype

The prevalence of SNHL did not differ among the three groups. Ocular phenotypes were not analyzed owing to data limitations.

## Discussion

This multicenter study in Korea allowed for the analysis of the genotype–phenotype correlation of male and female patients with XLAS who were diagnosed by genetic testing. Male patients in our study showed a significant difference in kidney survival between those with truncating variants and others, similar to the results of other studies^4–6,23^. In most previously reported studies^4–6,23^, cases with truncating variants developed CKD G5 earlier than cases with non-truncating variants. The kidney prognosis of male patients in our study was similar to that of a European study^5^. In contrast, the overall median age at CKD G5 in our study was earlier than that in the USA and Japanese cohort studies^4,23^ (Supplementary Table S3). The proportion of patients with missense variants, known to be associated with better prognosis, in our study (45%) was lower than that in the USA (64%) and Japanese (58%) cohort studies, and the kidney survival of our patients with missense variants (29 years) was shorter than that in the USA (37 years) and Japanese (40 years) studies as well. Although it is difficult to explain these differences, some possible reasons are as follows: Firstly, in our male patients with missense variants, RASi treatment was delayed approximately 6 years after the onset of renal symptoms (at a median age of 10.5 years) due to delayed hospital visits, and they already presented with significant proteinuria (the median UPCR of 1.5 mg/mg) at the first hospital visit. In a considerable number of cases in our study, RASi treatment was initiated when UPCR was ≥ 0.5 mg/mg for two or more consecutive measurements and discontinued when proteinuria improved after treatment. Recent guidelines recommend early and aggressive RASi use because it significantly prolongs kidney survival^24^. Secondly, our patients may have possessed missense variants resulting in a more severe phenotype. Kamura et al. suggested that poor trimerization or secretion defects could lead to a severe phenotype in missense mutations of the *COL4A5* gene^25^. None of the patients in our study had a genetic variant known as G624D, a common hypomorphic founder variant of European descent, or P628L and G1000V, which have been linked to mild phenotypes in previous studies^26–28^. Thirdly, the presence of modifier gene variants related to podocytes or the glomerular basement membrane may coexist and contribute to early-onset kidney failure^29^. Our study was limited as most patients were tested only using Sanger sequencing of the *COL4A5* gene, which may have resulted in the missed detection of modifier gene variants. Lastly, selection bias may have occurred as patients with a relatively severe phenotype were more likely to have been tested and included in this study.

Our study found that approximately one-fifth of male patients presented with NS as their first manifestation, which, to our knowledge, has not been well studied before. While most male patients (80–100%) present with microscopic hematuria, with or without macroscopic hematuria, proteinuria can be recognized at an early stage of childhood, sometimes leading to nephrotic syndrome^30^. In this study, we observed that NS was significantly more prevalent as the first manifestation in male patients with truncating variants. The higher incidence of NS at the initial visit could be associated with the higher rate of these variants. Furthermore, recent genetic studies have increasingly reported AS-causing genes in patients with steroid-resistant NS/focal segmental glomerulosclerosis^31^.

SNHL was detected in 65.1% of male patients in the present study; male patients with missense variants developed hearing problems significantly later than those with other variants, which is consistent with previous reports^4,5^. More than half of the patients with hearing loss required a hearing aid at the time of diagnosis, which means that the onset of hearing loss might be earlier. Hearing loss can occur even at preschool age in patients with severe variants^32^. Among female patients, 14.1% were found to have SNHL, which is higher than in the Japanese study (5.5%) but lower than in the European cohort (28%)^8,9^. The median age of the SNHL was 53.4 years, which is an earlier age than in the European cohort (more than 80 years). Despite these findings, there is limited research on hearing loss in females, so further studies are needed to better understand this condition. Moreover, early detection and proper management of SNHL are required to improve developmental, educational, and cognitive outcomes, particularly in children and adolescents^33^.

To date, two previous large-scale studies^8,9^ showed that female patients with XLAS, showed far more benign kidney outcomes compared to male patients: only 12–15% and 30–40% of patients developed CKD G5 by 40 and 60 years of age, respectively, without genotype–phenotype correlations. However, in a smaller study of 24 female patients with XLAS^34^, patients with non-missense variants showed a higher frequency of early proteinuria and a higher risk of developing CKD. Moreover, a recent systemic review^10^ provided more supportive evidence for a correlation between severe genotype and proteinuria, with proteinuria likely leading to kidney failure in female patients. The review found that proteinuria detected before 15 years of age progressed rapidly to kidney failure^10^ (Supplementary Table S4). In our study, more than 90% of the female patients developed overt proteinuria during follow-up, but the exact incidence at disease onset and onset date of proteinuria were unavailable. Despite RASi being used in nearly 90% of female patients at a median age of 9.5 years (approximately 5 years after onset presentation), 20.9% developed CKD G5 with a median age of 50.2 years (youngest age of 16.2 years). Kaplan–Meier analysis showed that over 30% and 60% of the patients progressed to CKD G5 by 40 and 60 years, respectively. In addition, our study showed a significant difference in kidney outcomes between female patients with non-truncating and truncating variants. Although the reasons for the relatively poorer kidney outcomes in our female patients compared to those in other studies are not clear, it is possible that a larger proportion of severe mutations, coexisting digenic variants, combined mutations such as those in other podocyte-related genes, and skewed X chromosome inactivation may have influenced phenotype severity in our patients^35,36^. Digenic AS, which occurs when variants in both *COL4A5* and C*OL4A3* or *COL4A4* genes are present, is known to have worse clinical features than a single variant in females^31,35^. Our study included more male patients than females, despite the fact that twice as many females as males are affected. It is possible that only women with more severe diseases were referred to the kidney clinic for evaluation, and limited genetic testing of patients with more severe phenotypes may have led to selection bias. Regarding hearing loss in female patients, there was no genotype–phenotype correlation, which is consistent with previous studies^8,9^.

Our study had several limitations. First, it was a retrospective study; thus, a major limitation was the absence of a unified RASi treatment protocol. Furthermore, there were considerable missing data points, especially for the affected family members. Second, the genetic testing in our study also posed some limitations, given that the test consisted only of Sanger sequencing of the *COL4A5* gene in most cases, with digenic and modifier gene variants not tested. Third, other exacerbating factors that might deteriorate kidney function, such as obesity and hypertension, could not be evaluated.

Nevertheless, this is the first multicenter study in Korea to investigate the genotype–phenotype correlation in a sizable number of males and females with XLAS. In particular, this is the first study to confirm the genotype–phenotype relationship between non-truncating and truncating variants in females with XLAS.

## Conclusions

In this multicenter retrospective study of Korean patients with XLAS, missense variants (mostly Gly substitutions) were the most common variant in both male (46.2%) and female (46.5%) patients. Despite using RASi in most patients, 57.9% of male and 20.9% of female patients developed CKD G5 at a median age of 25.0 and 50.2 years, respectively. Furthermore, SNHL was detected in 65.1% of male and 14.1% of female patients at a median age of 19.9 and 53.4 years, respectively. Among male patients, those with truncating variants had a shorter kidney survival period and higher frequency of SNHL than those with other types of variants (*P* < 0.001 in both). Even female patients with truncating variants had significantly poorer kidney survival than patients with other variants. These findings support the presence of genotype–phenotype correlation not only in male patients but also in female patients with XLAS.

## Supplementary Information

Supplementary Tables.

## Data Availability

The data that support the findings of this study are available from the corresponding author upon reasonable request.
