# Supplementary material for: Genotype–phenotype correlation of X-linked Alport syndrome observed in both genders: a multicenter study in South Korea
Source: Sci Rep. 2023 Apr 26;13:6827. doi: 10.1038/s41598-023-34053-7 (PMC10133262; doi:10.1038/s41598-023-34053-7)
Supplement: Supplementary file 1 — Supplementary Tables. [file 41598_2023_34053_MOESM1_ESM.docx]

Supplementary Table S1. List of 216 genetically proven X-linked Alport syndrome patients (124 probands) with kidney and hearing phenotypes.

| Patient number | Sex | Nucleotide change | Location | Protein change | Kidney outcome (age, years) | Hearing outcome (age, years) | Ocular findings | Novelty | Pathogenicity | ACMG criteria | |
| --- | --- | --- | --- | --- | --- | --- | --- | --- | --- | --- | --- |
| Missense (Glycine) mutations | | | | | | | | | | | |
| P1 | M | c.151G>A | Exon 3 | p.Gly51Arg | KFRT (28.8) | Normal (33.0) | NS | Novel | Likely pathogenic | | PP3, PP5, PM1, PM2 |
| P1-1 | M |  |  |  | KKFRT (18.6) | Normal (27.3) | NA |  |  | |  |
| P2 | M | c.404G>A | Exon 7 | p.Gly135Asp | CKD4 (23.1) | Normal (23.1) | Normal | Novel | Pathogenic | | PP3, PM1, PM6, PP5, PM2 |
| P3 | M | c.431G>A | Exon 7 | p.Gly144Asp | Normal (18.5) | Normal (18.5) | NA | Novel | Likely pathogenic | | PP3, PM1, PM2 |
| P3-1 | F |  |  |  | Normal (65.3) | Normal (65.3) | NA |  |  | |  |
| P4 | M | c.698G>T | Exon 13 | p.Gly233Val | Normal (20.4) | SNHL (20.9) | Normal | Known | Pathogenic | | PP3, PM1, PM5, PP5, PM2 |
| P5 | F | c.809G>A | Exon 14 | p.Gly270Glu | Normal (55.0) | Normal (55.0) | NA | Novel | Likely pathogenic | | PP3, PM1, PM2 |
| P6 | F | c.892G>A | Exon 16 | p.Gly298Ser | CKD4 (62.4) | SNHL (49.7) | NA | Known | Pathogenic | | PP3, PM1, PM2, PP5 |
| P7 | M | c.955G>C | Exon 17 | p.Gly319Arg | CKD3 (32.6) | SNHL (32.4) | NA | Known | Pathogenic | | PP3, PM1, PM5, PM2, PP5 |
| P7-1 | M |  |  |  | KFRT (24.0) | SNHL (NA) | NA |  |  | |  |
| P8 | F | c.1111G>C | Exon 19 | p.Gly371Arg | Normal (3.2) | Normal (3.2) | Normal | Known | Pathogenic | | PS1, PP3, PM1, PM5, PM6, PP5, PM2, BS4 |
| P9 | F | c.1121G>T | Exon 19 | p.Gly374Val | Normal (12.4) | Normal (12.4) | NA | Novel | Pathogenic | | PM5, PP3, PM1, PM2 |
| P9-1 | M |  |  |  | NA | SNHL (NA) | NA |  |  | |  |
| P10 | M | c.1138G>A | Exon 19 | P .Gly380Ser | Normal (15.4) | Normal (15.4) | Normal | Novel | Pathogenic | | PM5, PP3, PM1, PM6, PM2 |
| P11 | M | c.1199G>T | Exon 20 | p.Gly400Val | CKD4 (27.4) | Normal (27.4) | Normal | Novel | Likely pathogenic | | PP3, PM5, PM1, PM2 |
| P11-1 | F |  |  |  | NA | Normal (NA) | NA |  |  | |  |
| P12 | F | c.1199G>T | Exon 20 | p.Gly400Val | CKD2 (20.1) | Normal (20.1) | Normal | Novel | Likely pathogenic | | PP3, PM5, PM1, PM2 |
| P12-1 | M |  |  |  | KFRT (NA) | SNHL (NA) | NA |  |  | |  |
| P13 | F | c.1199G>T | Exon 20 | p.Gly400Val | KFRT (78.2) | SNHL (55.0) | NA | Novel | Likely pathogenic | | PP3, PM5, PM1, PM2 |
| P14 | F | c.1217G>T | Exon 20 | p.Gly406Val | Normal (19.9) | Normal (19.9) | NA | Known | Pathogenic | | PP3, PP5, PM1, PM5, PM2 |
| P14-1 | F |  |  |  | NA | Normal (NA) | NA |  |  | |  |
| P15 | M | c.1370G>T | Exon 21 | p.Gly457Val | Normal (6.6) | NA | NA | Novel | Pathogenic | | PM5, PP3, PM1, PM2 |
| P15-1 | F |  |  |  | NA | NA | NA |  |  | |  |
| P16 | M | c.1432G>C | Exon 22 | p.Gly478Arg | NA | Normal (19.9) | Normal | Novel | Likely pathogenic | | PP3, PM1, PM2 |
| P17 | M | c.1543G>T | Exon23 | p.Gly515Trp | normal(22.3) | SNHL(12.3) | Normal | Novel | Pathogenic | | PS2, PM5, PP3, PM1, PM2, PP1 |
| P18 | F | c.1580G>A | Exon 23 | p.Gly527Glu | Normal (18.5) | Normal (19.7) | Normal | Novel | Likely pathogenic | | PP3, PM1, PM2 |
| P18-1 | F |  |  |  | NA | Normal (NA) | NA |  |  | |  |
| P19 | M | c.1616G>A | Exon 24 | p.Gly539Asp | Normal (15.5) | Normal (15.5) | NA | Novel | Likely pathogenic | | PP3, PM1, PM2 |
| P19-1 | M |  |  |  | Normal (12.5) | Normal (12.5) | NA |  |  | |  |
| P19-2 | M |  |  |  | KFRT (19.0) | SNHL (NA) | NA |  |  | |  |
| P19-3 | F |  |  |  | NA | Normal (NA) | NA |  |  | |  |
| P20 | M | c.1903G>A | Exon 25 | p.Gly635Ser | KFRT (10.7) | Normal (13.5) | Normal | Novel | Likely pathogenic | | PM5, PP3, PM1, PM2, BS4 |
| P20-1 | F |  |  |  | Normal (42.0) | Normal (42.0) | NA |  |  | |  |
| P20-2 | F |  |  |  | Normal (11.0) | Normal (11.0) | NA |  |  | |  |
| P21 | F | c.1852G>C | Exon 25 | p.Gly618Arg | Normal (8.6) | Normal (8.6) | NA | Novel | Likely pathogenic | | PP3, PM1, PM6, PM2 |
| P22 | F | c.2095G>A | Exon 27 | p.Gly699Arg | Normal (6.6) | NA | NA | Novel | Likely pathogenic | | PP3, PM1, PM2 |
| P22-1 | M |  |  |  | NA | NA | NA |  |  | |  |
| P23 | F | c.2297G>T | Exon 29 | p.Gly766Val | CKD3 (53.4) | SNHL (53.4) | NA | Novel | Likely pathogenic | | PP3, PM1, PM2 |
| P23-1 | F |  |  |  | KFRT (44.0) | NA | NA |  |  | |  |
| P23-2 | F |  |  |  | Normal (21.0) | Normal (21.0) | Anisometropic amblyopia |  |  | |  |
| P23-3 | F |  |  |  | CKD2 (27.9) | Normal (27.9) | NA |  |  | |  |
| P24 | F | c.2332G>C | Exon 29 | p.Gly778Arg | CKD3 (40.5) | Normal (40.5) | NA | Known | Pathogenic | | PM5, PP3, PP5, PM1, PM2 |
| P24-1 | M |  |  |  | KFRT (42.0) | SNHL (32.0) | NA |  |  | |  |
| P25 | F | c.2351G>A | Exon 29 | p.Gly784Asp | Normal (11.4) | Normal (11.4) | NA | Novel | Likely pathogenic | | PP3, PM1, PM2 |
| P26 | M | c.2377G>A | Exon 29 | p.Gly793Arg | CKD3 (22.6) | Normal (22.6) | Normal | Novel | Pathogenic | | PM5, PP3, PM1, PM2 |
| P27 | M | c.2440G>A | Exon 30 | p.Gly814Arg | KFRT (38.1) | SNHL (47.9) | NS | Novel | Likely pathogenic | | PP3, PP5, PM1, PM2 |
| P27-1 | M |  |  |  | KFRT (33.0) | NA | NA |  |  | |  |
| P28 | M | c.2501G>A | Exon 30 | p.Gly834Asp | KFRT (24.7) | SNHL (16.7) | NA | Novel | Pathogenic | | PM5, PP3, PM6, PM1, PM2 |
| P29 | M | c.2510G>T | Exon 31 | p.Gly837Val | KFRT (15.0) | Normal (16.0) | Normal | Novel | Pathogenic | | PP3, PM1, PM2 |
| P29-1 | F |  |  |  | Normal (26.0) | Normal (26.0) | NA |  |  | |  |
| P30 | M | c.2605G>A | Exon 31 | p.Gly869Arg | CKD3 (16.4) | SNHL (15.8) | Normal | Known | Pathogenic | | PP5, PS1, PP3, PM1, PM5, PM2 |
| P31 | F | c.2669G>A | Exon 31 | p.Gly890Glu | Normal (10.0) | Normal (10.0) | Astigmatism, hyperopia, both | Novel | Likely pathogenic | | PP3, PM1, PM5, PM2 |
| P32 | M | c.2705G>A | Exon 32 | p.Gly902Glu | CKD4 (18.0) | Normal (18.0) | Normal | Novel | Likely pathogenic | | PP3, PM1, PM5, PM2 |
| P33 | M | c.2705G>T | Exon 32 | p.Gly902Val | NA | NA | NA | Known | Likely pathogenic | | PP3, PM1, PM2, PP5 |
| P34 | M | c.2732G>A | Exon 32 | p.Gly911Glu | KFRT (16.1) | NA | NA | Known | Likely pathogenic | | PP3, PM1, PM2, PP5 |
| P34-1 | M |  |  |  | KFRT (39.0) | NA | NA |  |  | |  |
| P35 | M | c.2767G>A | Exon 32 | p.Gly923Ser | KFRT (9.6) | SNHL (9.5) | NA | Novel | Pathogenic | | PP3, PM1, PM2 |
| P35-1 | F |  |  |  | NA | Normal (NA) | NA |  |  | |  |
| P36 | M | c.3035G>A | Exon 35 | p.Gly1012Asp | KFRT (37.5) | SNHL (27.5) | NA | Known | Pathogenic | | PP3, PP5, PM1, PM2 |
| P36-1 | M |  | Exon 35 |  | Normal (43.0) | SNHL (31.8) | Normal |  |  | |  |
| P37 | M | c.3152G>A | Exon 36 | p.Gly1051Glu | NA | NA | NA | Novel | Likely pathogenic | | PP3, PM5, PM1, PM2, PP5 |
| P38 | M | c.3161G>A | Exon 36 | p.Gly1054Asp | CKD2 (13.2) | SNHL (11.1) | Normal | Novel | Likely pathogenic | | PP3, PM1, PM2 |
| P38-1 | F |  |  |  | NA | Normal (NA) | NA |  |  | |  |
| P39 | M | c.3206G>T | Exon 36 | p.Gly1069Val | Normal (19.4) | Normal (19.4) | NA | Known | Pathogenic | | PP3, PP5, PM1, PM6, PM2 |
| P40 | M | c.3293G>T | Exon 37 | p.Gly1098Val | CKD3 (34.5) | NA | NA | Known | Pathogenic | | PP3, PM5, PM6, PM1, PM2 |
| P41 | M | c.3310G>C | Exon 37 | p.Gly1104Arg | KFRT (15.5) | SNHL (16.0) | NA | Novel | Pathogenic | | PM5, PP3, PM1, PM2, PP5 |
| P41-1 | F |  |  |  | Normal (21.4) | NA | NA |  |  | |  |
| P42 | F | c.3319G>A | Exon 37 | p.Gly1107Arg | KFRT (29.2) | Normal (29.2) | NA | Known | Pathogenic | | PP5, PS1, PP3, PM1, PM2 |
| P42-1 | F |  |  |  | KFRT (50.0) | NA | NA |  |  | |  |
| P43 | F | c.3428_3429delGCinsTA | Exon 38 | p.Gly1143Val | NA | NA | NA | Novel | Pathogenic | | PS1, PM1, PM5, PM2 |
| P43-1 | M |  |  |  | NA | NA | NA |  |  | |  |
| P44 | M | c.3508G>A | Exon 39 | p.Gly1170Ser | Normal (14.1) | Normal (14.1) | Normal | Known | Pathogenic | | PM5, PP3, PM1, PM2, PP5 |
| P44-1 | M |  |  |  | KFRT (35.0) | Normal (35.0) | NA |  |  | |  |
| P44-2 | F |  |  |  | Normal (46.4) | Normal (46.4) | Normal |  |  | |  |
| P45 | M | c.3622G>A | Exon 41 | p.Gly1208Arg | NA | NA | NA | Novel | Likely pathogenic | | PP3, PM1, PM5, PM2 |
| P46 | M | c.3686G>A | Exon 41 | p.Gly1229Asp | CKD3 (24.2) | Normal (24.2) | Normal | Known | Pathogenic | | PM5, PP3, PM1, PM2, PP5 |
| P46-1 | M |  |  |  | KFRT (13.0) | Normal (13.0) | NA |  |  | |  |
| P46-2 | M |  |  |  | KFRT (13.0) | Normal (13.0) | NA |  |  | |  |
| P46-3 | M |  |  |  | KFRT (24.0) | Normal (24.0) | NA |  |  | |  |
| P46-4 | M |  |  |  | KFRT (29.0) | Normal (29.0) | NA |  |  | |  |
| P47 | M | c.3800G>A | Exon 42 | p.Gly1267Asp | KFRT (18.0) | SNHL (19.9) | Normal | Novel | Likely pathogenic | | PP3, PM1, PM5, PM2 |
| P47-1 | F |  |  |  | NA | NA | NA |  |  | |  |
| P48 | M | c.3808G>C | Exon 42 | p.Gly1270Arg | Normal (24.4) | Normal (24.4) | Normal | Novel | Pathogenic | | PM5, PP3, PM1, PM2 |
| P49 | M | c.4324G>T, c.3170G>A | Exon 47  Exon 36 | p.Gly1442Cys, p.Gly1057Glu | CKD4 (29.2) | SNHL (10.5) | Normal | Novel, Novel | Pathogenic, likely Pathogenic | | PM5, PP3, PM1, PM2, PP3, PM1, PM5, PM2 |
| Missense (other than Glycine) mutations | | | | | | | | | | | |
| P50 | M | c.2330G>C | Exon 29 | p.Arg777Pro | Normal (13.2) | SNHL (11.7) | NS | Novel | Likely pathogenic | | PM1, PM2, PP3, PP4, BP1 |
| P50-1 | F |  |  |  | Normal (15.9) | Normal (15.9) | Normal |  |  | |  |
| P51 | M | c.2701A>G | Exon 32 | p.Met901Val | CKD4 (26.8) | Normal (26.8) | NA | Novel | Likely pathogenic | | PM1, PM2, PP3, PP4, BP1 |
| P52 | M | c.2938C>T | Exon 34 | p.Pro980Ser | KFRT (21.8) | SNHL (10.0) | Keratoconus | Novel | Likely pathogenic | | PM1, PM2, PP3, PP4, BP1 |
| P53 | M | c.4532G>A | Exon 48 | p.Arg1511His | KFRT (19.0) | SNHL (10.3) | Normal | Known | Likely pathogenic | | PM1, PM2, PP3, PP4, BP1 |
| P53-1 | M |  |  |  | KFRT (19.0) | NA | NA |  |  | |  |
| P54 | F | c.4706C>A | Exon 49 | p.Ala1569Asp | Normal (10.4) | Normal (10.4) | NA | Novel | Likely pathogenic | | PP3, PM6, PM1, PM2 |
| P55 | F | c.4632G>C | Exon 50 | p.Trp1544Cys | Normal (4.2) | Normal (4.2) | Normal | Novel | Likely pathogenic | | PP3, PM5, PM1, PM2 |
| P56 | M | c.5030G>T | Exon 51 | p.Arg1677Leu | Normal (25.2) | Normal (25.2) | NA | Novel | Pathogenic | | PM5, PP3, PM1, PM2 |
| P56-1 | M |  |  |  | Normal (22.7) | Normal (22.7) | NA |  |  | |  |
| P56-2 | M |  |  |  | Normal (13.5) | Normal (13.5) | Normal |  |  | |  |
| P56-3 | M |  |  |  | KFRT (35.0) | SNHL (NA) | NA |  |  | |  |
| P56-4 | M |  |  |  | KFRT (35.0) | SNHL (NA) | NA |  |  | |  |
| P56-5 | F |  |  |  | NA | Normal (NA) | NA |  |  | |  |
| In-frame short deletion or duplication mutations | | | | | | | | | | | |
| P57 | M | c.1365_1373del | Exon 21 | p.Pro458_Gly460del | KFRT (29.1) | SNHL (16.1) | NA | Novel | Likely pathogenic | | PM1, PM4, PM6, PM2 |
| P58 | F | c.4713_4721dup | Exon 49 | p.Val1572_lle1574dup | Normal (12.6) | SNHL (12.6) | Normal | Novel | Likely pathogenic | | PM1, PM4, PM2, PP4 |
| P58-1 | F |  |  |  | NA | Normal (NA) | NA |  |  | |  |
| Abnormal splice site mutations | | | | | | | | | | | |
| P59 | M | c.439-7A>G | Intron 7 |  | KFRT (17.2) | SNHL (18.6) | Normal | Novel | Uncertain Significance | | PP3, PM2, PP4 |
| P59-1 | M |  |  |  | Normal (11.8) | SNHL (NA) | Keratoconus, corneal hydrops |  |  | |  |
| P59-2 | M |  |  |  | KFRT (22.0) | NA | NA |  |  | |  |
| P59-3 | M |  |  |  | KFRT (17.0) | SNHL (NA) | NA |  |  | |  |
| P59-4 | F |  |  |  | Normal (12.1) | Normal (12.1) | Normal |  |  | |  |
| P60 | F | c.466-2A>G | Intron 8 |  | Normal (19.1) | NA | NA | Novel | Likely pathogenic | | PVS1, PM2 |
| P60-1 | M |  |  |  | NA | NA | NA |  |  | |  |
| P60-2 | F |  |  |  | NA | NA | NA |  |  | |  |
| P61 | F | c.466-2A>G | Intron 8 |  | Normal (8.4) | Normal (8.4) | NA | Novel | Pathogenic | | PVS1, PM2, PP1 |
| P61-1 | F |  |  |  | Normal (31.9) | Normal (31.9) | NA |  |  | |  |
| P61-2 | F |  |  |  | CKD2 (22.1) | Normal (22.1) | NA |  |  | |  |
| P61-3 | M |  |  |  | CKD4 (31.1) | SNHL (22.4) | NA |  |  | |  |
| P61-4 | M |  |  |  | KFRT (25.0) | SNHL (NA) | NA |  |  | |  |
| P62 | F | c.645+1G>A | Intron 11 |  | Normal (21.9) | SNHL (8.5) | NA | Novel | Likely pathogenic | | PVS1, PM2 |
| P63 | F | c.834+1G>C | Intron 14 |  | Normal (31.4) | NA | NA | Novel | Pathogenic | | PVS1, PM2, PP5 |
| P63-1 | M |  |  |  | KFRT (44.1) | SNHL (40.1) | Anterior lenticonus, fleck retinopathy |  |  | |  |
| P63-2 | M |  |  |  | KFRT (42.1) | SNHL (NA) | Abnormal, NA |  |  | |  |
| P63-3 | M |  |  |  | KFRT (34.1) | SNHL (NA) | Abnormal, NA |  |  | |  |
| P63-4 | M |  |  |  | KFRT (39.1) | SNHL (NA) | Abnormal, NA |  |  | |  |
| P64 | M | c.835-2A>C | Intron 14 |  | KFRT (38.8) | Normal (43.6) | Normal | Novel | Likely pathogenic | | PVS1, PM2 |
| P65 | M | c.936+1G>A | Intron 16 |  | KFRT (33.2) | SNHL (23.0) | NA | Novel | Pathogenic | | PVS1, PP5, PM2 |
| P65-1 | F |  |  |  | Normal (11.2) | Normal (11.2) | Normal |  |  | |  |
| P66 | M | c.991-7T>A | Intron 17 |  | Normal (19.3) | Normal (19.3) | Normal | Novel | Uncertain Significance | | PP3, PM2, PP4 |
| P67 | F | c.991-7T>A | Intron 17 |  | Normal (19.0) | Normal (19.0) | Normal | Novel | Uncertain Significance | | PP3, PM2, PP4 |
| P68 | M | c.1032+3_1032+6del | Intron 18 |  | Normal (8.6) | Normal (8.6) | NS | Known | Pathogenic | | PP5, PP3, PM2 |
| P68-1 | F |  |  |  | KFRT (35.0) | Normal (35.0) | NA |  |  | |  |
| P68-2 | F |  |  |  | Normal (13.8) | Normal (13.8) | Normal |  |  | |  |
| P68-3 | F |  |  |  | Normal (9.9) | Normal (9.9) | Normal |  |  | |  |
| P69 | M | c.1165+1G>A | Intron 19 |  | KFRT (16.9) | SNHL (11.5) | Normal | Novel | Pathogenic | | PVS1, PM6, PM2 |
| P70 | M | c.1340-2A>G | Intron 20 |  | KFRT (35.4) | SNHL (NA) | Cataract | Novel | Likely pathogenic | | PVS1, PM2 |
| P70-1 | M |  |  |  | KFRT (29.5) | Normal (29.5) | Normal |  |  | |  |
| P71 | F | c.1588-1G>T | Intron 23 |  | KFRT (24.6) | Normal (24.6) | NA | Novel | Likely pathogenic | | PVS1, PM2 |
| P72 | M | c.1779+3G>C | Intron 24 |  | Normal (13.6) | SNHL (12.5) | Normal | Novel | Likely pathogenic | | PP3, PM6, PM2 |
| P73 | M | c.1779+4_1779+8del | Intron 24 |  | KFRT (24.7) | SNHL (15.1) | Fleck macular deposit, both | Novel | Uncertain Significance | | PP3, PM2, PP4 |
| P74 | M | c.2244+1G>A | Intron 28 |  | KFRT (21.4) | SNHL (10.0) | Macular degeneration and temporal inner retinal thinning, both &  Decreased vision | Novel | Likely pathogenic | | PVS1, PM2 |
| P74-1 | M |  |  |  | KFRT (25.0) | NA | NA |  |  | |  |
| P74-1 | F |  |  |  | KFRT (55.3) | SNHL (42.2) | NA |  |  | |  |
| P75 | M | c.2509+2_2509+5del | Intron 30 |  | Normal (14.8) | SNHL (9.5) | NA | Novel | likely pathogenic | | PVS1, PM2 |
| P75-1 | F |  |  |  | NA | NA | NA |  |  | |  |
| P76 | M | c.2678-3A>G | Intron 31 |  | KFRT (13.3) | SNHL (9.2) | Normal | Novel | Uncertain Significance | | BP4, PM2, PP4 |
| P76-1 | M |  |  |  | CKD2 (14.1) | SNHL (9.4) | Normal |  |  | |  |
| P77 | F | c.3247-1G>C | Intron 36 |  | CKD2 (21.6) | Normal (21.6) | NA | Novel | Pathogenic | | PVS1, PM6, PM2 |
| P78 | M | c.3247-1G>A | Intron 36 |  | Normal (17.4) | SNHL (13.6) | Normal |  | Pathogenic | | PVS1, PM2, PP5 |
| P78-1 | F |  |  |  | NA | Normal (NA) | NA |  |  | |  |
| P79 | M | c.4298-8G>A | Intron 46 |  | CKD2 (13.2) | Normal (13.2) | Normal | Novel | Uncertain Significance | | PP3, PM2, PP4 |
| P79-1 | M |  |  |  | Normal (18.9) | Normal (18.9) | Normal |  |  | |  |
| P79-2 | F |  |  |  | NA | Normal (NA) | NA |  |  | |  |
| P80 | M | c.4297+1G>T | Intron 46 |  | Normal (16.0) | Normal (16.0) | Normal | Novel | Likely pathogenic | | PVS1, PM2 |
| P81 | M | c.2678-1G>T | Intron 31 |  | Normal (5.5) | Normal (5.5) | Normal | Novel | Pathogenic | | PVS1, PS2, PM2, PP1 |
| Nonsense mutations | | | | | | | | | | | |
| P82 | F | c.59G>A | Exon 1 | p.Trp20Ter | Normal (2.8) | Normal (2.8) | NA | Novel | Pathogenic | | PVS1, PS2, PM2, PP1 |
| P83 | M | c.544C>T | Exon 9 | p.Gln182Ter | KFRT (9.4) | SNHL (5.4) | Myopic astigmatism | Novel | Pathogenic | | PVS1, PM6, PM2 |
| P84 | F | c.667C>T | Exon 12 | p.Gln223Ter | KFRT (16.2) | Normal (16.2) | Normal | Novel | Pathogenic | | PVS1, PS2, PM2, PP1 |
| P85 | M | c.703C>T | Exon 13 | p.Gln235Ter | CKD2 (21.7) | SNHL (NA) | Peripheral retinal degeneration, both | Novel | Pathogenic | | PVS1, PM6, PM2 |
| P86 | F | c.796C>T | Exon 14 | p.Arg266Ter | CKD2 (17.2) | SNHL (NA) | NS | Known | Pathogenic | | PVS1, PP5, PM6, PM2 |
| P87 | M | c.960C>G | Exon 17 | p.Tyr320Ter | KFRT (17.2) | SNHL (10.3) | Corneal erosion, Lt & Minimal cataract, both & Decreased vision | Novel | Pathogenic | | PVS1, PM6, PM2 |
| P88 | F | c.1117C>T | Exon 19 | p.Arg373Ter | Normal (9.7) | Normal (9.7) | NA | Known | Pathogenic | | PVS1, PP5, PM2 |
| P89 | F | c.1933C>T | Exon 25 | p.Gln645Ter | Normal (5.1) | Normal (5.1) | Normal | Known | Pathogenic | | PVS1, PP5, PM2 |
| P90 | F | c.2731G>T | Exon 32 | p.Gly911Ter | Normal (3.6) | Normal (3.6) | NA | Novel | Likely pathogenic | | PVS1, PM2 |
| P90-1 | F |  |  |  | NA | Normal (NA) | NA |  |  | |  |
| P91 | M | c.3112C>T | Exon 36 | p.Gln1038Ter | NA | SNHL (NA) | Normal | Novel | Pathogenic | | PVS1, PM6, PM2 |
| P92 | F | c.3250G>T | Exon 37 | p.Glu1084Ter | Normal (10.8) | NA | NA | Novel | Likely pathogenic | | PVS1, PM2 |
| P93 | M | c.3904C>T | Exon 42 | p.Gln1302Ter | NA | NA | NA | Novel | Pathogenic | | PVS1, PP5, PM2 |
| P93-1 | M |  |  |  | KFRT (20.0) | NA | NA |  |  | |  |
| P93-2 | M |  |  |  | KFRT (20.0) | NA | NA |  |  | |  |
| P94 | F | c.4078G>T | Exon 45 | p.Gly1360Ter | Normal (18.7) | Normal (18.7) | Normal | Novel | Likely pathogenic | | PVS1, PM2 |
| P94-1 | F |  |  |  | NA | Normal (NA) | NA |  |  | |  |
| P95 | M | c.4099C>T | Exon 45 | p.Gln1367Ter | CKD3 (18.3) | SNHL (11.0) | Normal | Novel | Likely pathogenic | | PVS1, PM2 |
| P95-1 | M |  |  |  | KFRT (17.0) | SNHL (NA) | NA |  |  | |  |
| P95-2 | F |  |  |  | NA | SNHL (NA) | NA |  |  | |  |
| P96 | M | c.4687C>T | Exon 48 | p.Arg1563Ter | KFRT (12.3) | SNHL (14.6) | Corneal erosion, Rt | Known | Pathogenic | | PVS1, PP5, PM2 |
| P97 | M | c.5020C>T | Exon 51 | p.Arg1674Ter | KFRT (26.6) | SNHL (25.5) | NA | Known | Pathogenic | | PVS1, PP5, PM2 |
| P97-1 | F |  |  |  | KFRT (30.0) | SNHL (NA) | NA |  |  | |  |
| P98 | M | c.5029C>T | Exon 51 | p.Arg1677Ter | CKD2 (20.7) | SNHL (8.6) | Corneal erosion | Known | Pathogenic | | PVS1, PM6, PP5, PM2 |
| P99 | F | c.5029C>T | Exon 51 | p.Arg1677Ter | Normal (7.1) | Normal (7.1) | NA | Known | Pathogenic | | PVS1, PP5, PM2 |
| P99-1 | F |  |  |  | NA | NA | NA |  |  | |  |
| P100 | F | c.5029C>T | Exon 51 | p.Arg1677Ter | Normal (11.0) | Normal (11.0) | NA | Known | Pathogenic | | PVS1, PM6, PP5, PM2 |
| Short frameshifting mutations | | | | | | | | | | | |
| P101 | M | c.176del | Exon 3 | p.Pro59GlnfsTer96 | KFRT (19.4) | Normal (20.2) | NA | Novel | Likely pathogenic | | PVS1, PM2 |
| P101-1 | F |  |  |  | CKD3 (24.0) | Normal (24.0) | NA |  |  | |  |
| P102 | M | c.358_367delinsTGGAACCTGGT | Exon 6 | p.Gly120TrpfsTer38 | Normal (9.8) | SNHL (9.3) | NA | Novel | Likely pathogenic | | PVS1, PM2 |
| P103 | M | c.546del | Exon 10 | p.Gly183AlafsTer20 | KFRT (24.8) | SNHL (6.8) | Corneal erosion, recurrent keratitis | Novel | Likely pathogenic | | PVS1, PM2 |
| P104 | F | c.738_747del | Exon 13 | p.Glu246AspfsTer5 | Normal (5.2) | Normal (5.2) | NA | Novel | Likely pathogenic | | PVS1, PM2 |
| P105 | M | c.958del | Exon 17 | p.Tyr320ThrfsTer26 | CKD3 (23.9) | SNHL (NA) | NS | Novel | Pathogenic | | PVS1, PS2, PM2, PP1 |
| P106 | M | c.1013del | Exon 18 | p.Pro338HisfsTer8 | KFRT (20.9) | SNHL (14.2) | NA |  | Likely pathogenic | | PVS1, PM2 |
| P107 | M | c.1967del | Exon 26 | p.Gly656ValfsTer4 | CKD3 (17.5) | SNHL (14.9) | Normal | Novel | Likely pathogenic | | PVS1, PM2 |
| P107-1 | M |  |  |  | KFRT (20.0) | SNHL (NA) | NA |  |  | |  |
| P108 | F | c.2322del | Exon 29 | p.Gly775ValfsTer17 | Normal (10.2) | Normal (10.2) | NA | Novel | Likely pathogenic | | PVS1, PM2 |
| P109 | M | c.2600dup | Exon 31 | p.Gly869ArgfsTer29 | Normal (12.9) | SNHL (8.5) | Astigmatism | Novel | Pathogenic | | PVS1, PM6, PM2 |
| P110 | M | c.2777_2781del | Exon 33 | p.Gly926AlafsTer15 | KFRT (15.7) | SNHL (15.7) | Normal | Novel | Likely pathogenic | | PVS1, PM2 |
| P110-1 | M |  |  |  | KFRT (25.0) | NA | NA |  |  | |  |
| P110-2 | F |  |  |  | KFRT (23.0) | NA | NA |  |  | |  |
| P110-3 | F |  |  |  | NA | NA | NA |  |  | |  |
| P111 | M | c.2915del | Exon 33 | p.Pro972GlnfsTer24 | Normal (13.7) | SNHL (10.1) | Normal | Novel | Likely pathogenic | | PVS1, PM2 |
| P112 | F | c.3458del | Exon 39 | p.Gly1153ValfsTer146 | Normal (24.5) | Normal (24.5) | NA | Novel | Likely pathogenic | | PVS1, PM2 |
| P113 | M | c.3672del | Exon 41 | p.Glu1224AspfsTer75 | KFRT (20.6) | SNHL (8.3) | NA | Novel | Likely pathogenic | | PVS1, PM2 |
| P113-1 | F |  |  |  | KFRT (50.2) | Normal (50.2) | NS |  |  | |  |
| P114 | M | c.3914del | Exon 42 | p.Pro1305GlnfsTer15 | KFRT (17.3) | SNHL (10.3) | Normal | Known | Pathogenic | | PVS1, PP5, PM2 |
| P114-1 | M |  |  |  | KFRT (20.0) | NA | NA |  |  | |  |
| P115 | M | c.3875_3876TA>C | Exon 42 | p.Leu1292ProfsTer7 | Normal (13.2) | Normal (13.2) | NA | Novel | Likely pathogenic | | PVS1, PM2 |
| P116 | M | c.4045del | Exon 44 | p.Glu1349AsnfsTer22 | KFRT (25.6) | SNHL (10.5) | NS | Known | Pathogenic | | PVS1, PP5, PM2 |
| P116-1 | M |  |  |  | KFRT (28.0) | NA | NA |  |  | |  |
| P117 | M | c.4088del | Exon 45 | p.Gly1363ValfsTer8 | KFRT (12.6) | SNHL (9.6) | Perifoveal retinal thinning, both | Novel | Pathogenic | | PVS1, PP5, PM2 |
| P117-1 | F |  |  |  | KFRT (26.6) | Normal (26.6) | NA |  |  | |  |
| P117-2 | F |  |  |  | KFRT (37.0) | Normal (37.0) | NA |  |  | |  |
| P118 | M | c.4490del | Exon 47 | p.Arg1497LysfsTer51 | KFRT (20.6) | SNHL (20.0) | NA | Novel | Likely pathogenic | | PVS1, PM2 |
| P118-1 | F |  |  |  | KFRT (39.0) | Normal (39.0) | NA |  |  | |  |
| P119 | M | c.4731_4732delCA | Exon 49 | p.His1577GlnfsTer62 | KFRT (24.9) | SNHL (25.4) | Anterior lenticonus | Known | Pathogenic | | PVS1, PP5, PM2 |
| P119-1 | F |  |  |  | CKD3 (29.0) | Normal (29.0) | NA |  |  | |  |
| P120 | M | c.275_276+2del | Exon 4 + intron 4 | | CKD3 (24.0) | Normal (24.0) | NA | Novel | Likely pathogenic | | PVS1, PM2 |
| P120-1 | M |  |  |  | CKD2 (16.5) | NA | NA |  |  | |  |
| P121 | M | c.1940_1948+5del | Exon 25 + intron 25 | | CKD2 (15.0) | NA | NA | Novel | Likely pathogenic | | PVS1, PM2 |
| Large frameshifting mutations | | | | | | | | | | | |
| P122 | M | Exon 22-28 deletion | Exon 22-28 | | KFRT (29.7) | SNHL (19.2) | Cataract | Unknown |  | |  |
| P123 | M | Total deletion |  |  | KFRT (26.3) | SNHL (7.3) | Corneal opacity | Unknown |  | |  |
| P124 | M | Total deletion |  |  | KFRT (25.0) | SNHL (11.5) | NA | Unknown |  | |  |
| P124-1 | M |  |  |  | KFRT (16.2) | SNHL (10.5) | NA |  |  | |  |
| P124-2 | F |  |  |  | NA | SNHL (21.2) | NA |  |  | |  |

The patients with numbers connected by dash are affected family members with the same mutation as the corresponding proband

ACMG, American College of Medical Genetics; M, male; F, female; KFRT, kidney failure with replacement therapy; CKD, chronic kidney disease; NA, not available; NS, non-specific; SNHL, sensorineural hearing loss; Sanger, sanger sequencing, TES, targeted exome sequencing; WES, whole exome sequencing; Rt, right; Lt, left; BRVO, branch retinal vein occlusion; CRVO, central retinal vein occlusion; PACS, primary angle closure suspects

* Stage of CKD are defined as follows: G2, eGFR 60-89; G3, eGFR 30-59; G4, eGFR 15-29. eGFR, estimated glomerular filtration rate in mL/min/1.73m^2^, is calculated using the creatinine-cystatin C-based Schwart 2012 equation.

* Pathogenicity according to ACMG guideline: pathogenic criterion; very strong (PVS1), strong (PS1-4), moderate (PM1-6), or supporting (PP1-5), and benign criterion; strong (BS1-4), or supporting (BP1-6).

**Supplementary Table S2. Variants classified as uncertain significance.**

| **Patient**  **number** | **Sex** | **Nucleotide change** | **Frequency in gnomAD** | **In-silico prediction** | | | | **Family segregation** | **Family history** | **Kidney biopsy** | |
| --- | --- | --- | --- | --- | --- | --- | --- | --- | --- | --- | --- |
|  |  |  |  | **SpliceAI** | **HSF^a^** | **MaxEnt^a^** | **CADD** |  |  | **GBM change in EM** | **IF stain** |
| P66 | M | c.991-7T>A^b^ | 0 | AG 0.96  AL 0.26 | 43.5/71.4  (64.1%) | -4.44/3.93  (188.5%) | 22.3 | NA | Mother: HU  Two maternal uncles: CKD | Diffuse abnormalities including focal thinning, thickening, and reticulation | Alpha 5 chain loss |
| P67 | F | c.991-7T>A^b^ | 0 | AG 0.96  AL 0.26 | 43.5/71.4  (64.1%) | -4.44/3.93  (188.5%) | 22.3 | Paternal germline | Father: CKD | Diffuse thinning, multifocal reticulation, and splitting | ND |
| P59 | M | c.439-7A>G | 0 | AG 0.60  AL 0.85 | 62.6/90.5  (44.5%) | -0.64/8.11  (1367.2%) | 25.8 | Maternal germline | Mother: HU  Brother: AS  Maternal male cousin: AS | Thinning and thickening with irregular contour | ND |
| P73 | M | c.1779+4_1779+8del | 0 | DG 0.64  DL 0.73 | 80.5/56.3  (-30.0%) | 7.21/-12.32  (-270.9%) | 25.0 | NA | Mother: HU, PU, and SNHL | Irregular thinning, thickening, and splitting | ND |
| P76 | M | c.2678-3A>G | 0 | AG 0.42  AL 0.93 | 55.3/83.1  (50.4%) | -3.20/5.55  (273.4%) | 24.4 | Maternal germline | Mother: HU  Brother: AS | Diffuse abnormalities including focal thinning, thickening, lamellation, and reticulation | Alpha 5 chain loss |
| P79 | M | c.4298-8G>A | 0 | AG 0.90  AL 0.75 | 54.6/82.4  (51.1%) | -2.68/5.28  (297.0%) | 21.6 | Maternal germline | Mother: HU  Brother: AS | Thinning, splitting, and lamellation with irregular contour | ND |

gnomAD, genome aggregation database; HSF, Human Splicing Finder; CADD, Combined Annotation-Dependent Depletion; GBM, glomerular basement membrane; EM, electron microscopy; IF, immunofluorescence; AG, acceptor gain; AL, acceptor loss; NA, not available; HU, hematuria; CKD, chronic kidney disease; ND, not done; AS, Alport syndrome; DG, donor gain; DL, donor loss; PU, proteinuria; SNHL, sensorineural hearing loss.

^a^The results are presented as reference score/mutation score (variation %)

^b^An in-vitro analysis using mRNA sequencing was recently conducted for another family who had the same variant. It revealed a splicing effect which corresponds to the retention of 5 bp in intron 17 at position r.990_991insaacag in approximately 80% of transcripts. As a result, the variant was reclassified as likely pathogenic.

**Supplementary Table S3. Comparison of kidney survival periods in this study with those from previously reported studies in male patients with X-linked Alport syndrome**

| Study | Nation | Total | Missense | Splice | Truncating |
| --- | --- | --- | --- | --- | --- |
| Jais JP et al.^4^ | Europe | 25 (NA), n=315 | 32 (NA),  n=124 (39%) | 25 (NA),  n=54 (17%) | 21 (NA),  n=137 (44%) |
| Bekheirnia et al.^3^ | United states | NA,  n=681 | 37 (34–40),  n=438 (64%) | 28 (26–32),  n=78 (12%) | 25 (21–31),  n=70 (10%) |
| T Yamamura et al.^23^ | Japan | 35 (32–40),  n=422 | 40 (35–45),  n=245 (58%) | 28 (23–34)  n=69 (16%) | 18 (16–27), n=30 (7%)^a^  21 (19–55), n=14 (3%)^b^ |
| This study | Korea | 25 (24–27),  n=120 | 29 (24–35),  n=54 (45%) | 30 (21–38),  n=29 (24%) | 21 (15–26),  n=37 (31%) |

Kidney survival periods (years) are presented as median (95% confidence interval), estimated using the Kaplan-Meier method.

NA, not available.

**^a^**Nonsense variant

**^b^**Large rearrangement

**Supplementary Table S4. Comparison of kidney survival periods in this study with those from previously reported studies in female patients with X-linked Alport syndrome**

| Study | Nation | Total | Missense | Splice | Truncating |
| --- | --- | --- | --- | --- | --- |
| Jais JP et al.^8^ | Europe | > 80, n=288 | >80, n=115 (40%) | NA, n=46 (16.0%) | >80, n=127 (44.1%) |
| T Yamamura et al.^9^ | Japan | 65, n=250 | 65, n=129 (52%) | 65, n=43 (17%) | 66, n=23 (9%)^a^ |
| Gibs JT et al.^10^ | Systemic  review | 65, n=305 | 65, n=172 (56%) | 59, n=56 (18%) | 66, n=77 (25%) |
| This study | Korea | 50, n=68 | 78, n=30 (44%) | 55, n=16 (24%) | 37, n=22 (32%) |

Kidney survival periods (years) are presented as the median estimated using the Kaplan-Meier method.

NA, not available.

^a^Nonsense variants
